# Effect of thawing methods on frozen semen quality of yak (*Poephagus grunniens L*.) bulls

**DOI:** 10.14202/vetworld.2015.831-834

**Published:** 2015-07-07

**Authors:** Binod Kumar Dutta Borah, Bharat Chandra Deka, Ranjan Kumar Biswas, Prithiviraj Chakravarty, Sourabh Deori, Sudip Sinha, Kutubuddin Ahmed

**Affiliations:** 1 ICAR-National Research Centre on Yak, Dirang-790101, Arunachal Pradesh, India; 2 Department of Animal Reproduction, Gynaecology and Obstetrics, College of Veterinary Science, Assam Agricultural University, Khanapara, Guwahati -781022, Assam, India

**Keywords:** acrosomal changes, post-thaw, thawing methods, semen quality, yak

## Abstract

**Aim:**

To evaluate different thawing temperatures and duration on the post-thaw semen quality of Indian yaks bulls.

**Materials and Methods:**

Semen ejaculates from four different yak bulls were collected using artificial vagina method and extended with tris extender containing 6.4% glycerol at 35°C, cooled gradually from 35°C to 5°C at 1°C/3 min and equilibrated at 4-5°C for 4 h and frozen in French mini straws using a programmable bio-freezer and finally stored in liquid nitrogen. Thawing of frozen semen straws was carried out using three methods i.e., 35°C for 60 s (thawing method I), 37°C for 30 s (thawing method II) and 75°C for 9 s (thawing method III). The post-thaw semen quality parameters assessed were sperm motility, percent live sperm, hypo-osmotic swelling test (HOST)-reacted sperm, acrosomal changes, and alanine aminotransferase (ALT) and aspartate aminotransferase (AST) activities in the extracellular media.

**Results:**

The percent sperm motility, total incidence of acrosomal changes, and extracellular release of AST varied significantly (p<0.01) between thawing methods but live sperm and HOST-reacted sperm did not vary significantly between thawing methods. The percent sperm motility of frozen yak semen for thawing method III was significantly (p<0.05) higher than that for thawing methods I and II, the difference between thawing methods I and II being non-significant. The critical difference test revealed that the total incidence of acrosomal changes and extracellular release of AST were significantly (p<0.05) lower when thawing was done using methods I and II than in method III.

**Conclusion:**

On the basis of the present experiment, we can conclude that barring the post-thaw sperm motility, thawing of frozen yak semen in water either at 35°C for 60 s or 37°C for 30 s gives better post-thaw semen quality than at 75°C for 09 s.

## Introduction

Thawing of frozen semen brings back the frozen spermatozoa to life and to the physiologically active state. Therefore, it is essential to carry out thawing carefully at an optimal temperature for sufficient time to minimize the loss of semen quality during thawing procedure.

Worldwide, different workers conducted trials to determine the optimal thawing temperature and duration, and to know the adequate thawing rate that may result in the highest percentage of viable spermatozoa in different species after thawing process [1,2]. However, such study was not reported for Indian yak bull’s semen.

In India, yak is distributed throughout the 3000 km of snow-bound Himalayan range starting from the Kashmir Valley of Northwest India to Arunachal Pradesh in the east. Standardization of artificial insemination technique in this species can go a long way for genetic dissemination of superior germplasm under field conditions and *ex situ* conservation of this valuable highland species.

Therefore, the present study was undertaken with an objective to evolve a suitable thawing protocol for yak bull semen and its effect on post-thaw semen quality.

## Materials and Methods

### Ethical approval

This study was conducted after approval by the Research Committee and Institutional Animal Ethics Committee.

### Semen collection and processing

Semen ejaculates were collected by artificial vagina from apparently healthy yak bulls aged 3-4.5 years, maintained at the yak farm of the ICAR – National Research Centre on Yak, Dirang, Arunachal Pradesh, India. The bulls were maintained under similar managerial conditions and fed with concentrate mixture comprising maize grain, wheat bran, mustard cake, groundnut cake, soyabean meal, mineral mixture, and salt at 2-2.5 kg/bull/day along with seasonal green grasses and tree leaves *ad libitum*.

Immediately after collection, the semen was evaluated for color, volume, mass activity, and initial sperm motility using standard methods. A total of 15 ejaculates having a minimum volume of 1.00 ml, mass activity 3+ and initial sperm motility 70% were used for processing and freezing. The semen samples were extended (1:10) with tris extender containing 6.4% glycerol at 35°C, cooled gradually from 35°C to 5°C at 1°C/3 min and equilibrated at 4-5°C for 4 h. Following equilibration, freezing of semen in French mini straw was done using a programmable bio freezer (Mini- Digitcool, IMV Technology, France) as per Contri *et al*. [3] and semen straws were plunged into the liquid nitrogen and stored.

Thawing of frozen semen straws was carried out in water using three different methods i.e., 35°C for 60 s (thawing method I), 37°C for 30 s (thawing method II), and 75°C for 9 s (thawing method III). The post-thaw semen quality parameters assessed were sperm motility, percent live sperm, hypo-osmotic swelling test (HOST)-reacted sperm as described by Jeyendran *et al*. [4], acrosomal changes using Giemsa staining technique of Watson [5], and alanine aminotransferase (ALT) and aspartate aminotransferase (AST) activities in the extracellular media estimated by enzymatic colorimetric method using commercial ENZOKIT (RFCL Limited, Plot No. 37, Pharma City, Selaqui, Dehradun - 248197, Uttarakhand, India) and were expressed in IU/L.

### Statistical analysis

Analyses were performed using the Statistical System software package (SAS, Cary, NC, USA, 2010). Data on different parameters were analyzed using two-factorial analysis of variance (ANOVA) (GLM procedure).

## Results

The mean percentage of motile sperm, live sperm, HOST-reacted sperm, extracellular ALT (IU/L), AST (IU/L), and different acrosomal changes in frozen semen after thawing in water following thawing method I, II, and III are presented in Table-1.

**Table-1:** Effect of different thawing methods on various post-thaw sperm parameters.

Parameters	Thawing methods		

	I (35°C for 60 s)	II (37°C for 30 s)	II (75°C for 09 s)
Sperm motility (%)	64.45^A^±0.83	64.67^A^±0.91	66.33^B^±1.03
Live sperm (%)	70.05±0.73	70.40±0.78	69.87±0.41
HOSTreacted sperm (%)	57.00±1.04	59.45±1.22	58.00±1.20
ALT (IU/L)	11.87±0.77	11.95±1.02	12.38±0.92
AST (IU/L)	11.37^A^±0.65	10.28^A^±0.10	14.39^B^±0.74
Swollen acrosome (%)	5.50^A^±0.37	5.10^A^±0.19	6.60^B^±0.32
Separating acrosome (%)	2.33±0.23	2.33±0.19	2.80±0.20
Entirely lost acrosome (%)	3.47±0.31	3.87±0.19	3.73±0.34
Total acrosomal changes (%)	11.00^A^±0.62	11.40^A^±0.27	13.27^B^±0.53

A,B,C Means bearing different letter superscripts differ significantly (p<0.05). HOST=Hypo-osmotic swelling test, ALT=Alanine aminotransferase, AST=Aspartate aminotransferase

The percent sperm motility (p<0.01) varied significantly between thawing methods but live sperm and HOST-reacted sperm did not vary significantly between thawing methods. Critical difference test revealed that the percent sperm motility of frozen yak semen for thawing method III was significantly (p<0.05) higher than that for thawing methods I and II, but difference between thawing methods I and II was non-significant. The extracellular release of AST differed significantly (p<0.01) between thawing methods but, ALT did not differ significantly between thawing methods. The extracellular release of AST for thawing methods I and II was significantly (p<0.05) lower than that for thawing method III.

Analysis of variance revealed that the incidences of the swollen acrosome and total acrosomal changes differed significantly (p<0.01) between thawing methods but the incidence of separating and entirely lost acrosomes did not differ significantly between thawing methods. The critical difference test revealed that the incidence of the swollen acrosome and total incidence of acrosomal changes were significantly (p<0.05) lower when thawing was done using methods I and II than in method III.

## Discussion

In the present study, the percent sperm motility in frozen yak semen was found to be significantly (p<0.05) higher for thawing method III than that for thawing methods I and II. Similar to the present findings, significantly (p<0.05) higher post-thaw sperm motility in buffalo semen was reported by earlier workers for thawing at 75°C for 9 s than at 37°C for 15 s [6,7] and 37°C for 30 s [8]. The post-thaw motility and kinematic parameters of buffalo spermatozoa were significantly improved immediately after thawing by increasing the thawing rate from 37°C in 30 s to70°C in 6 s [9]. In bull semen, Lyashenko [10] recorded that progressive motility was significantly increased by thawing at 65-70°C for 6-7 s compared with the thawing rate of 35°C for 20 s. Nema *et al*. [11] also observed significantly higher sperm motility in semen samples thawed at 50°C for 8 s than that thawed at 40°C for 8 s or at 37°C for 8 or 15 s. The higher sperm motility observed when semen was thawed at higher temperature might be due to that even the weakly motile spermatozoa were moving at a higher velocity. Higher (60°C) thawing temperatures were also preferred by other workers [12,13]. The post-thaw sperm motility did not differ significantly when semen was thawed at 35°C for 60 s or at 37°C for 30 s.

In the present study, although not significant, the percentage of live sperm in frozen yak semen was recorded apparently higher for thawing method I and II in comparison to method III. On the contrary, Ahmed [8] obtained a higher percentage of live sperm when frozen buffalo semen was thawed at 75°C for 9 s in comparison with that thawed at 37°C for 30 s. The above discrepancies might be due to the differences in processing and freezing protocols used in different studies and the species involved.

The percentage of HOST-reacted sperm was also apparently higher for thawing methods I and II than for thawing method III, but the difference was not significant. Similar observations were also reported by Ahmed [8] in frozen buffalo semen and Sarmah [14] in frozen buck semen. No significant difference in the percentage of HOST-reacted sperm was recorded when thawed at 37°C for 30 s, 25°C for 2 min, 50°C for 12 s, and 75°C for 9 s was reported by Ahmed [8] and between 37°C for 30 s, 37°C for 2 min, and 40°C for 20 s was by Sarmah [14].

The total incidence of acrosomal changes in yak semen was significantly (p<0.05) lower for thawing methods I and II than that for thawing method III. Although not significant, the extracellular release of ALT was slightly lower when semen was thawed using thawing methods I and II as compared to thawing method III. On the other hand, the extracellular release of AST in frozen semen was found to be significantly (p<0.05) lower when semen was thawed following the method I and II than III. The lower extracellular release of ALT and AST in frozen semen thawed at 35°C for 60 s and at 37°C for 30 s could be due to lower injuries inflicted to cell membrane as evidenced by significantly lower total incidence of acrosomal changes recorded in the study.

The thawing phase of frozen semen is equally important to that of freezing phase as both the stages are to pass through the critical temperature zone i.e., from −5 to −50°C [15] which causes detrimental effect on surviving spermatozoan population. The lipid peroxidation and subsequent membrane damage are at its peak in the frozen-thawed semen [16]. The effect of thawing depends on the rate of freezing, i.e., whether the rate of freezing has been sufficiently high to induce intracellular freezing or slow enough to produce cell dehydration. In the former, fast thawing is required to prevent recrystallization of any intracellular ice present in the spermatozoa. Spermatozoa thawed at a faster rate may be exposed for a shorter time to the concentrated solute and glycerol, and the restoration of the intracellular and extracellular equilibrium is more in rapid thawing than in slow thawing [12,17]. The differences observed in the present study and those indicated by other workers might be explained by the differences in pairing of the freezing rate and thawing method. However, it was recommended that for optimal cell survival, the rate at which the cell is frozen must be matched with a corresponding thaw rate, to reverse the osmotic balance and rehydration of the cell, while preventing intracellular ice formation [18].

## Conclusion

Barring the post-thaw sperm motility, thawing of frozen yak semen in water either at 35°C for 60 s or 37°C for 30 s gives better post-thaw semen quality than at 75°C for 9 s as evident by significantly lesser (p<0.05) incidence of swollen acrosome, total acrosomal changes, and extracellular AST release. The percentage of live sperm, HOST-reacted sperm, extracellular ALT release, and incidence of separating acrosome was also recorded better; however, the differences were non-significant.

## Authors’ Contributions

BKDB carried out the research work as a part of his Ph. D thesis. BCD and PC conceptualized and formulated the experimental design. SD supervised the experiment and drafted the manuscript. RKB, SS, and KA analyzed the data and revised the manuscript. All authors read and approved the manuscript.
